# Mechanisms of Cellular Senescence: Cell Cycle Arrest and Senescence Associated Secretory Phenotype

**DOI:** 10.3389/fcell.2021.645593

**Published:** 2021-03-29

**Authors:** Ruchi Kumari, Parmjit Jat

**Affiliations:** MRC Prion Unit at UCL, UCL Institute of Prion Diseases, London, United Kingdom

**Keywords:** cellular senescence, cell cycle arrest, senescence associated secretory phenotype (SASP), DNA damage response (DDR), DREAM complex

## Abstract

Cellular senescence is a stable cell cycle arrest that can be triggered in normal cells in response to various intrinsic and extrinsic stimuli, as well as developmental signals. Senescence is considered to be a highly dynamic, multi-step process, during which the properties of senescent cells continuously evolve and diversify in a context dependent manner. It is associated with multiple cellular and molecular changes and distinct phenotypic alterations, including a stable proliferation arrest unresponsive to mitogenic stimuli. Senescent cells remain viable, have alterations in metabolic activity and undergo dramatic changes in gene expression and develop a complex senescence-associated secretory phenotype. Cellular senescence can compromise tissue repair and regeneration, thereby contributing toward aging. Removal of senescent cells can attenuate age-related tissue dysfunction and extend health span. Senescence can also act as a potent anti-tumor mechanism, by preventing proliferation of potentially cancerous cells. It is a cellular program which acts as a double-edged sword, with both beneficial and detrimental effects on the health of the organism, and considered to be an example of evolutionary antagonistic pleiotropy. Activation of the p53/p21^WAF1/CIP1^ and p16^INK4A^/pRB tumor suppressor pathways play a central role in regulating senescence. Several other pathways have recently been implicated in mediating senescence and the senescent phenotype. Herein we review the molecular mechanisms that underlie cellular senescence and the senescence associated growth arrest with a particular focus on why cells stop dividing, the stability of the growth arrest, the hypersecretory phenotype and how the different pathways are all integrated.

## Introduction

Cellular senescence, a seminal discovery of Hayflick and Moorhead (1961) is a process that globally regulates cell fate and can be considered a hallmark of aging (Hayflick and Moorhead, 1961; López-Otín et al., 2013). Hayflick demonstrated that upon serial passaging, normal human diploid fibroblast cell strains cease to divide *in vitro* after a fixed number (40–60) of population doublings, the Hayflick limit (Hayflick and Moorhead, 1961).

Senescence is triggered by developmental signals or different kinds of stress. Depending on the cell type and intensity and nature of the stress, cells may respond by inducing repair, cell death or senescence (Surova and Zhivotovsky, 2013; Galluzzi et al., 2018; Sapieha and Mallette, 2018). Cells can undergo senescence in response to various intrinsic and extrinsic stimuli, including progressive telomere shortening, changes in telomeric structure, mitogenic signals, oncogenic activation, radiation, oxidative and genotoxic stress, epigenetic changes, chromatin disorganization, perturbed proteostasis, mitochondrial dysfunction, inflammation, and/or tissue damage signals, irradiation, or chemotherapeutic agents, nutrient deprivation (Di Micco et al., 2006; Kuilman et al., 2010; Passos et al., 2010; Pazolli et al., 2012; García-Prat et al., 2016; Mikuła-Pietrasik et al., 2020).

These different types of stress signals give rise to different types of senescence such as telomere dependent replicative senescence, programmed senescence or non-telomeric stress-induced premature senescence including oncogene-induced senescence (OIS), unresolved DNA damage induced senescence, epigenetically induced senescence and mitochondrial dysfunction associated senescence (Toussaint et al., 2002; Debacq-Chainiaux et al., 2016). An extensive study by Petrova et al. (2016) identified more than 50 small chemical compounds that can induce premature senescence and senescence-like states. Recent studies have demonstrated that treatment with some anticancer agents, chemotherapeutic drugs or ionizing radiation provoke “therapy-induced senescence (TIS)” in tumor cells (Di Micco et al., 2006; Ewald et al., 2010; Dörr et al., 2013; Toso et al., 2014; Petrova et al., 2016; Dabrowska et al., 2018; Saleh et al., 2018, 2019; Mikuła-Pietrasik et al., 2020).

Senescence is now considered to be a highly dynamic, multi-step process, during which the properties of senescent cells continuously evolve and diversify in a context dependent manner (Van Deursen, 2014; Boisvert et al., 2018). It is associated with multiple cellular, molecular changes and distinct phenotypic alterations including a stable and generally irreversible proliferation arrest unresponsive to mitogenic stimuli. Senescent cells remain viable with alterations in metabolic activity and are usually resistant to apoptosis (Wang, 1995; Hampel et al., 2004; Marcotte et al., 2004; Ryu et al., 2007; Sanders et al., 2013; Childs et al., 2014; Zhu et al., 2015). They undergo dramatic gene expression changes along with chromatin remodeling and engagement of a persistent DNA damage response (DDR) (Rodier et al., 2009; Wang et al., 2009; Sulli et al., 2012; Chandra et al., 2015). One characteristic feature of senescent cells is increased lysosomal activity (Kurz et al., 2000; Lee et al., 2006), macromolecular damage (Gorgoulis et al., 2019), and a temporal cascade in the development of the complex senescence-associated secretory phenotype (SASP) (Coppé et al., 2008, 2010a; Saleh et al., 2018). Senescent cells can also develop morphological and structural changes, including an enlarged, flattened, multinucleated morphology with enlarged vacuoles (Campisi and D’Adda Di Fagagna, 2007), altered composition of the plasma membrane and a remarkable nuclear enlargement (Kuilman et al., 2010; Salama et al., 2014; Frescas et al., 2017; Hernandez-Segura et al., 2018; Ramos et al., 2020). These complex changes to the cell serve to implement various aspects of senescence such as growth arrest and the development of SASP secretome.

Initially senescence was thought to be a tissue culture artifact. However, multiple subsequent studies have demonstrated the importance of senescence in different physiological and pathological processes (Burton and Krizhanovsky, 2014). Senescence plays key physiological roles in normal development (Muñoz-Espín et al., 2013; Storer et al., 2013), maintaining tissue homeostasis, tissue remodeling and repair (Yun et al., 2015), wound healing (Ramakrishna et al., 2012; Demaria et al., 2014), secretion of insulin by pancreatic beta cells (Helman et al., 2016), and limits tumor progression by ensuring that potentially dysfunctional, damaged or transformed cells do not perpetuate their genomes to the next generation (Collado et al., 2007; Hanahan and Weinberg, 2011; Kang et al., 2015; Childs et al., 2017; Maciejowski and De Lange, 2017; Faget et al., 2019; Wang et al., 2020).

Senescent cells have been found to accumulate exponentially with increasing chronological age in multiple tissues (Muñoz-Espín and Serrano, 2014; Hudgins et al., 2018). The early work of Hayflick and Moorhead (1961) for the first time hinted toward a relationship between senescence and aging, but subsequent discoveries have demonstrated the presence of senescent cells *in vivo* and an increase in their number with age supporting the hypothesis that senescence itself can drive aging and is one of its key hallmarks (Hayflick and Moorhead, 1961; Hayflick, 1965; López-Otín et al., 2013).

Cellular senescence also has deleterious effects as it can hinder tissue repair and regeneration and contribute to tissue and organismal aging due to the accumulation of senescent cells and depletion of stem/progenitor cell compartments and secretion of SASP (Coppé et al., 2010a; Campisi et al., 2011). Senescent cells have been observed in several age-related diseases such as atherosclerosis, diabetes, lung disease, and many others (Muñoz-Espín and Serrano, 2014; Chandrasekaran et al., 2017; McHugh and Gil, 2018). Although senescence is associated with aging, cells can undergo senescence irrespective of organismal age due to different signals apart from telomere shortening. In accordance with this the use of transgenic mouse models have allowed the detection of senescent cells in different age related pathologies and enabled the development of genetic or pharmacological strategies to demonstrate that selective elimination of senescent cells can prevent or delay age-related tissue dysfunction to extend life span and improve health span (Baker et al., 2011; Xu et al., 2015a; Baker et al., 2016; Hashimoto et al., 2016; Zhao et al., 2018).

Cellular senescence is a cellular program which acts as a double-edged sword with both beneficial and detrimental effects on the health of the organism, and thereby considered to be an example of evolutionary antagonistic pleiotropy (Williams, 1957; Kirkwood and Austad, 2000; Campisi, 2003; Giaimo and D’Adda di Fagagna, 2012; Ohtani et al., 2012; Schosserer et al., 2017).

Taken together, senescence is both a physiologically fundamental and pathologically relevant program, with its role depending on the context and the specific situation. Here, we review the different mechanisms controlling cellular senescence with a special focus on cell cycle arrest and SASP. We detail the complexity of the mechanisms involved in SASP regulation, focus on the key mediators, characteristic hallmarks and the different pathways involved in manifesting cellular senescence as well as the cell cycle arrest and its key regulators along with the role of the DREAM complex and its associated components. The significance of cellular senescence in different contexts such as its role *in vivo*, in cancer and aging are also discussed. At the end we discuss the translational relevance and suitability for identifying and characterizing senescent cells *in vivo* to explore potential future avenues for exploiting the benefits and preventing the detrimental aspects of senescent cells such as suppressing the SASP or selectively eliminating senescent cells to increase health span.

## Senescence Mediated Cell Cycle Arrest

The cell cycle is a sequence of coordinated events which lead to cell division, critical for both development and viability of multicellular organisms. A stable cell cycle arrest which marks an inability of the cell to continue dividing is an indispensable and one of the defining features of senescent cells. Cell cycle arrest can be an alarm response instigated by aberrant proliferation or deleterious stress stimuli to prevent the propagation of dysfunctional cells.

Cellular senescence is different from another form of growth arrest known as quiescence, in that senescence occurs in G1 and possibly G2 phase of the cell cycle (Di Leonardo et al., 1994) as opposed to quiescence which happens in G0. Another crucial difference is that quiescent cells can resume proliferation in response to appropriate signals such as stimulation by growth factors or mitogenic signals whereas senescent cells cannot (Campisi and D’Adda Di Fagagna, 2007; Calcinotto et al., 2019; Gorgoulis et al., 2019; Mohamad Kamal et al., 2020). This is beautifully explained by Blagosklonny’s theory of ‘hyperfunction’ which states that aging is a quasi-program, that occurs as a consequence of processes occurring during development and growth in early life (Blagosklonny, 2013). For example, during growth arrest, the nutrient sensing pathways like mTOR (mechanistic target of Rapamycin) remain active but now as opposed to cell proliferation and growth, this initiates cellular senescence. Therefore, the choice between senescence and quiescence is governed to a certain extent by the mTOR pathway. Cells with persistent activation of mTOR undergo a stable senescent growth arrest, whereas cells undergo quiescence when mTOR is inhibited (Korotchkina et al., 2010; Blagosklonny, 2012). Apoptosis is a programmed cell death in which the remains of a dead apoptotic cell are removed by engulfment by another cell, whereas in senescence, the senescent cell is not immediately eliminated and remains metabolically active despite being in an arrested state. Senescence is also different from terminally differentiated cells which have also irreversibly withdrawn from the cell cycle wherein undifferentiated precursor cells are converted into specialized effector cells (Baumann, 2016; Hinze and Boucrot, 2018; Mohamad Kamal et al., 2020). All together distinct signaling pathways are involved in terminal differentiation such as Notch, Wingless and Hedgehog (Gorgoulis et al., 2019). Terminally differentiated cells can also undergo cellular senescence showing that senescence does not depend on an active cell cycle (Jurk et al., 2012; von Zglinicki et al., 2020). Although the cell cycle arrest in cellular senescence is believed to be irreversible, studies have recently shown that senescent cells can under certain circumstances re-enter the cell cycle such as in tumor cells (Galanos et al., 2016; Patel et al., 2016; Milanovic et al., 2018; Saleh et al., 2019) or be reprogrammed into induced pluripotent stem cells (Banito and Gil, 2010; Lapasset et al., 2011).

**FIGURE 1:**
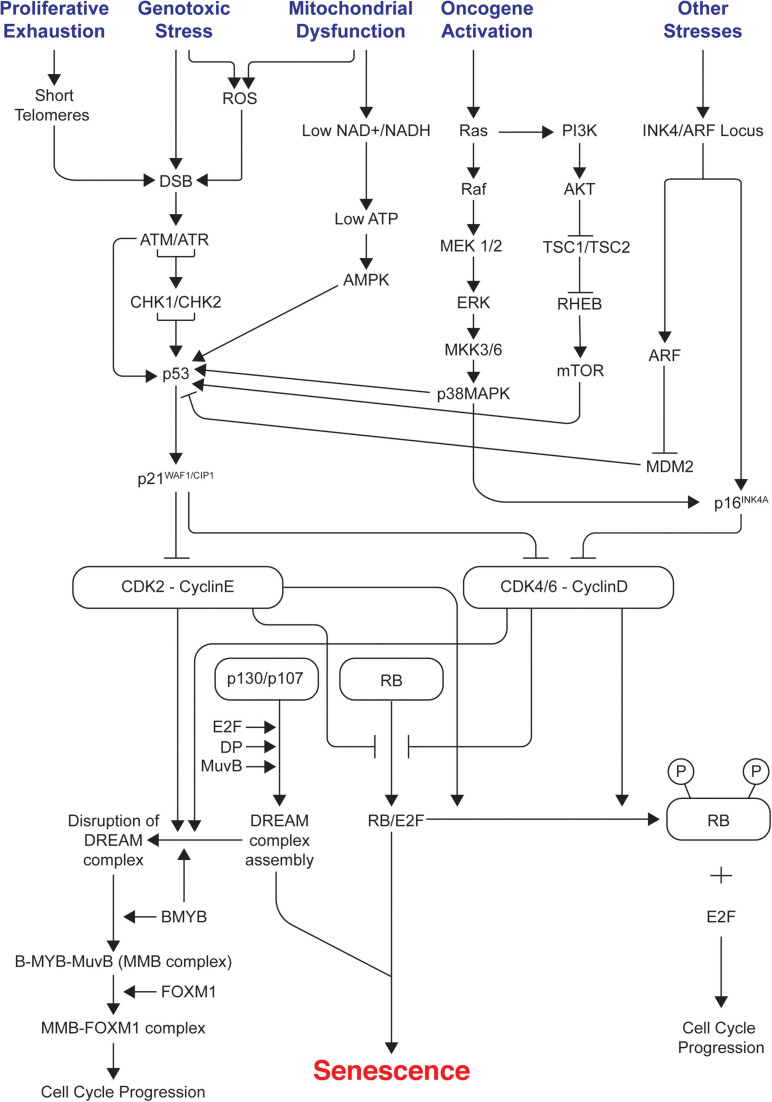
Signals and pathways involved in mediating senescence mediated cell cycle arrest. The figure shows the different intrinsic and extrinsic stimuli capable of inducing cellular senescence. Key pathways involved in manifesting cell cycle arrest in senescence such as p53/p21^WAF1/CIP1^ and p16^INK4A^/RB tumor suppressor pathways, DDR, AMPK, p38/MAPK, PI3K/AKT/mTOR are illustrated. It indicates how different pathways are interconnected and how the assembly of repressive DREAM complex triggers senescence and its disruption leads to cell cycle progression. ROS, reactive oxygen species; DDR, DNA damage response; DREAM, dimerization partner (DP), RB-like, E2F, and MuvB core complex.

Cell cycle arrest in senescence is largely mediated via activation of either one or both p53/p21^WAF1/CIP1^ and p16^INK4A^/pRB tumor suppressor pathways (Rovillain et al., 2011; Kobashigawa et al., 2019; Liu and Wan, 2019). Both these pathways are complex as they involve many upstream regulators and downstream effectors along with varying side branches (Chau and Wang, 2003; Levine and Oren, 2009). Both pathways are also interlinked with extensive crosstalk (Martín-Caballero et al., 2001; Zhang et al., 2006; Yamakoshi et al., 2009). They maintain the senescence state mainly by inducing widespread changes in gene expression as p53 and pRB are key transcriptional regulators; p21^WAF1/CIP1^ acts downstream of p53 whereas p16^INK4A^ acts upstream of pRB. They are the crucial components of each pathway as they are cyclin-dependent kinase inhibitors (CDKIs) and act as negative regulators of cell cycle progression. Prolonged overexpression of any of these four critical components (p53, pRB, p16^INK4A^, p21^WAF1/CIP1^) is sufficient to induce senescence (McConnell et al., 1998). Figure 1 summarizes the different signals and pathways involved in mediating senescence mediated cell cycle arrest.

### p53/p21^WAF1/CIP1^ Pathway

p53/p21^WAF1/CIP1^ is activated in response to DNA damage caused by telomere attrition, oxidative or oncogenic stress. Constitutive DNA damage response (DDR) signaling leads to chronic activation of p53 which induces cellular senescence. Inactivation of p53 mediated signaling by a variety of approaches can disrupt the onset of cellular senescence (Shay et al., 1991; Beauséjour et al., 2003).

p53, famously known as the ‘Guardian of the genome’ plays a key role in manifestation of cellular senescence via several different mechanisms (Lane, 1992; Kastenhuber and Lowe, 2017). Activation of p53 is dependent on various post translational modifications such as phosphorylation, methylation, acetylation, sumoylation, ubiquitination, and neddylation (Kruse and Gu, 2009). Increased Ser-15 phosphorylation by Ataxia Telengectasia Mutated (ATM) kinase results in p53 stabilization and was found to be the only common change between replicative senescence and DNA damage induced senescence (Webley et al., 2000). Activated p53 regulates expression of a set of anti-proliferative genes (Olivier et al., 2010; Kastenhuber and Lowe, 2017).

As p53 performs different functions within a cell, it is regulated at multiple different levels by different factors. MDM2, an E3 ubiquitin ligase regulates the levels of p53 in conjunction with MDM4. Interaction of p53 with FOXO4 during cellular senescence plays a crucial role in regulating its transcriptional activity and localization (Baar et al., 2017). The signaling mediated by FOXO and its target protein 4E-BP regulates aging in Drosophila by removing damaged proteins thereby delaying muscle function decay and extending life span (Demontis and Perrimon, 2010).

p21^WAF1/CIP1^, a 21 KDa protein encoded by the CDKN1A gene, is a member of the Cip/Kip family of CDKIs in addition to p27 and p57. It is capable of inactivating all CDKs, thereby inhibiting cell cycle progression (Wade Harper et al., 1993). It inhibits the kinase activity of cyclin-CDK complexes by interacting with cyclins through the two cyclin binding motifs (Cy1 and Cy2). This leads to inhibition of phosphorylation of the RB family of proteins and subsequent association with E2Fs and formation of the DREAM complex thereby leading to a cell cycle arrest (Chen et al., 1996; Whittaker et al., 2017; Wiedemeyer, 2018). p21^WAF1/CIP1^ plays a dual conflicting role in cell cycle progression depending on its level of expression (Al Bitar and Gali-Muhtasib, 2019). High levels of p21^WAF1/CIP1^ inhibit the kinase activity of cyclinD/CDK4,6 complexes leading to inhibition of cell cycle progression whereas low levels of p21^WAF1/CIP1^ act as an assembly factor for cyclinD/CDK4,6 complex and promote its activation resulting in cell cycle progression (Labaer et al., 1997; Welcker et al., 1998; Cheng et al., 1999; Deng et al., 2018). p21^WAF1/CIP1^ was the first identified transcriptional target for p53 (El-Deiry et al., 1993). As it is known to interact with and inactivate various cyclin/CDK complexes, it is capable of inducing cell cycle arrest at any stage of the cell cycle as opposed to the INK4 family of CDKIs which specifically bind and inactivate CDK4 and CDK6, thereby inducing a cell cycle arrest only during G0/G1 phase (Wade Harper et al., 1993; Pavletich, 1999; Sherr, 2000). p21^WAF1/CIP1^ can also be activated by p53 independent mechanisms by other stimulators such as nuclear receptors including androgen, vitamin D and retinoid receptors. Members of the Krüppel-like factor (KLF) transcription factor (TF) family can activate the *CDKN1A* gene by cooperating with p300-CREBBP (Aliouat-Denis et al., 2005; Abbas and Dutta, 2009).

Induction of p21^WAF1/CIP1^ is crucial for initiation of senescence mediated growth arrest by different stimuli (Noda et al., 1994; Hernandez-Segura et al., 2017). Upregulation of p21^WAF1/CIP1^ plays a key role in developmental senescence as mice lacking it show defects in embryonic senescence, apical ectodermal ridge maintenance and patterning as well as other developmental defects. Developmental senescence is a transient programmed cellular senescence that occurs during mammalian embryonic development (Muñoz-Espín et al., 2013; Storer et al., 2013). However, expression of p21^WAF1/CIP1^ does not persist in senescent cells as it is mainly required for induction of senescence (Stein et al., 1999; Sharpless and Sherr, 2015; He and Sharpless, 2017; Song et al., 2020). In contrast p16^INK4A^ is required to maintain the senescent state.

In addition to the transcriptional control of p21^WAF1/CIP1^ by p53 dependent and independent mechanisms, it is also regulated at the post translational level. Newly synthesized p21^WAF1/CIP1^ is stabilized by WISp39, a Hsp90 binding tetratricopeptide repeat protein, that prevents its proteasome mediated degradation (Jascur et al., 2005). Additional post-translational modifications such as phosphorylation can modulate binding partners or change the subcellular location, which has the potential to alter its function by blocking its ability to act as a CDKI (Child and Mann, 2006). p21^WAF1/CIP1^, when present within the nucleus, inhibits cell cycle progression, whereas upon phosphorylation it gets transported to the cytoplasm where it functions as an anti-apoptotic protein (Ping et al., 2006). Therefore, p21^WAF1/CIP1^ plays multiple roles within the cell by regulating different processes (Karimian et al., 2016; Georgakilas et al., 2017).

Transient stress leads to induction of p53 which activates DNA repair and leads to quiescence (Vousden and Prives, 2009; Kasteri et al., 2018). Cells can resume proliferation upon resolution of the stress (Childs et al., 2015). Additional signals and persistent stress can lead to sustained expression of p53 and activation of p16^INK4A^ contributing to a long lasting cell cycle arrest (Sharpless and DePinho, 2006; Salama et al., 2014; Kruiswijk et al., 2015). Cell fate is determined by different factors which is further complicated by the context dependent role of p16^INK4A^ and p21^WAF1/CIP1^.

p53 is known to indirectly downregulate expression of many factors required for cell cycle progression. Essentially all the genes are downregulated indirectly by p53 as only about 3% of them are directly bound by it (Fischer et al., 2014). Repression by p53 involves direct activation of p21^WAF1/CIP1^ leading to formation of different repressive complexes such as RB/E2F and DREAM (Fischer et al., 2014, 2016a, 2016b; Fischer and Müller, 2017).

### p16^INK4A^/pRB Pathway

The RB family of pocket proteins is one of the main targets of cyclin-CDK complexes and their best-known function is binding to and inactivating E2F complexes leading to repression of E2F target gene transcription. There are three members of the RB pocket protein family: RB1 (pRB), RBL1 (p107), and RBL2 (p130). These proteins share a common bipartite pocket region comprising a LXCXE motif, which allows them to interact directly with other proteins (Dyson, 1998).

pRB when dephosphorylated binds to E2Fs thereby forming a repressive RB-E2F complex. These repressive complexes bind to the promoter regions of E2F target genes and inhibit the transcription of genes required for cell cycle progression (Fischer and Müller, 2017). To enhance transcription repression, they recruit factors such as histone deacetylases (HDACs) and the histone methyltransferase SUV39H1. At the restriction point this inhibition is removed by hyperphosphorylation of RB by cyclinE-CDK2 which leads to release of E2Fs, thereby promoting transcription of S phase genes and hence progression of the cell cycle (Zhang et al., 2000). It has been suggested that crosstalk between RB and mitogenic AKT signaling pathways play a key role in the quiescence to senescence switch by regulating overlapping functions of Forkhead transcription factors, FOXO3a and FOXM1 (Eijkelenboom and Burgering, 2013; Lam et al., 2013; Imai et al., 2014). Moreover Argonaute (AGO2), microRNA (let-7) and RB1 interact in the nucleus to repress certain E2F target genes such as CDC2 and CDCA8 during senescence (Benhamed et al., 2012).

INK4/ARF locus encodes three tumor suppressors namely p16^INK4A^ and p14^ARF^ encoded by CDKN2A gene and p15^INK4B^ by CDKN2B gene (Sharpless, 2005; Gil and Peters, 2006). Similarly, to p21^WAF1/CIP1^, p15^INK4B^, and p16^INK4A^ are CDKIs and affect cell cycle progression by binding to and inhibiting CDK4/6. In contrast, p14^ARF^ establishes cross talk between the p53 and pRB pathways by regulating the stability of p53 by binding to and inhibiting MDM2, responsible for its proteasome-mediated degradation (Gil and Peters, 2006; Kim and Sharpless, 2006). Expression of ARF is regulated by p53 via a negative feedback loop (Kotake et al., 2011).

p16^INK4A^ is a 16 KDa protein that directly binds to CDK4/6 and blocks the formation of cyclinD-CDK4/6 complexes, thereby preventing phosphorylation of RB and promoting expression of E2F target genes (Serrano et al., 1993). This crucial role is evident from the fact that loss of the p16^INK4A^ gene or inherited mutations within it have been frequently related to several human cancers particularly malignant melanoma (Gil and Peters, 2006; Kim and Sharpless, 2006; Li et al., 2011). This suggests that inactivation or loss of p16^INK4A^ leads to bypass of senescence, thereby promoting cancer.

Epigenetically induced senescence mostly acts by inducing p16^INK4A^ expression as opposed to DNA damage-induced senescence which relies mainly on p21^WAF1/CIP1^ (Petrova et al., 2016). Since epigenetic modifiers are capable of maintaining the senescent state without inducing any cell stress, epigenetically induced senescence has been characterized as ‘causeless’ which makes it similar to the senescence observed during development or upon aging as opposed to DNA damage-induced senescence which occurs prematurely due to induction of different forms of stress (Petrova et al., 2016).

Replicative senescence is also linked to derepression of the CDKN2A locus. In young tissues, the CDKN2A locus is normally expressed at a very low undetectable level whereas it becomes derepressed leading to a high-level of expression upon aging (Krishnamurthy et al., 2004). The molecular mechanisms underlying this derepression are not completely understood but have been associated with loss of polycomb group of proteins but independent of p53 (Jacobs et al., 1999; Bracken et al., 2007).

Polycomb proteins are a group of conserved proteins required to maintain stable repression of specific target genes by histone modification (Gould, 1997; Van Lohuizen, 1998). Polycomb protein complexes such as PRC1/PRC2 silence the INK4/ARF locus (Martin et al., 2014). Therefore, p16^INK4A^ mediated senescence can be induced via disruption of PRC1/PRC2 complex components such as CBX7, BMI1 or EZH2 followed by decrease in levels of H3K27me3 (Jacobs et al., 1999; Bracken et al., 2003, 2007; Gil et al., 2004). Epigenetic regulation of the INK4/ARF locus is not limited to polycomb proteins as other epigenetic regulators such as ZRF, MLL1 or JMJD3 are also involved in its regulation (Gil and Peters, 2006; Agger et al., 2009; Barradas et al., 2009; Kotake et al., 2009; Ribeiro et al., 2013). Polycomb group members such as CBX7, EED, EZH2 and SUZ12 are downregulated by microRNAs miR-26b, miR-181a, miR-210, and miR-424, which leads to p16^INK4A^ activation and hence senescence induction (Overhoff et al., 2014).

The epigenetic alterations occurring during senescence are quite diverse and cell type and context dependent. Alterations in DNA methylation are observed during replicative senescence whereas cells undergoing oncogene induced senescence do not show any such alterations in DNA methylation (Cruickshanks et al., 2013; Cheng et al., 2017; Xie et al., 2018).

Cell cycle progression can also be affected by TGF-β as it maintains RB in a hypophosphorylated state, thereby inducing cell cycle arrest in lung epithelial cells in G1 (Laiho et al., 1990). In addition to p16^INK4A^, p15^INK4B^ has been shown to an effector of TGF-β meditated cell cycle arrest by inhibiting CDK4/6 (Hannon and Beach, 1994).

### DREAM Complex Mediated Cell Cycle Arrest

Sadasivam and DeCaprio (2013) described the DREAM complex as the master coordinator of cell cycle-dependent gene expression. DREAM is a multi-subunit complex formed by the assembly of p130 and p107 (RB family of pocket proteins) with Dimerization partner (DP), E2F4-5 and a Multivulval class B (MuvB) core complex which represses most if not all gene expression in quiescence (Litovchick et al., 2007). The MuvB core complex comprises LIN9, LIN37, LIN52, LIN54, and RBBP4 (Sadasivam et al., 2012), originally identified in *Caenorhabditis elegans* (Fay and Yochem, 2007).

During G0 all cell cycle dependent gene expression is repressed by binding of the DREAM complex. It has been shown that in mammalian cells, phosphorylation of p130 leads to dissociation of the DREAM complex resulting in the MuvB core complex recruiting B-MYB to activate late S-phase genes, and FOXM1, in G2 phase, to activate mitotic gene expression (Litovchick et al., 2007; Schmit et al., 2007; Sadasivam et al., 2012). Since the DREAM complex binds to cell cycle genes homology region (CHR) promoter elements in addition to E2F binding sites (Schmit et al., 2009; Müller and Engeland, 2010; Müller et al., 2012), it has the potential to regulate a larger set of genes than RB and perform distinct regulatory functions apart from RB/E2F complexes (Müller et al., 2012; Guiley et al., 2015; Fischer and Müller, 2017). Assembly of the DREAM complex also requires phosphorylation of the LIN52 component of the MuvB core complex at Serine-28 (Litovchick et al., 2011). Even though the role of the DREAM complex in cellular senescence is not fully understood, it has been shown that disorganization of DREAM complex by ectopic expression of a non-phosphorylatable LIN52 leads to suppression of Ras-induced senescence (Litovchick et al., 2011; Iness et al., 2019).

Initially, the detailed mechanism by which p53 mediates transcriptional repression of a plethora of genes was not understood, however this changed after the availability of genome-wide ChIP data on p53 binding sites and the discovery of the mammalian DREAM complex along with its target genes (Litovchick et al., 2007; Schmit et al., 2007). This has led to the observation that p53 induction leads to the formation of the repressive DREAM complex and the identification of the p53-DREAM pathway (Quaas et al., 2012). The discovery of p21^WAF1/CIP1^-DREAM-E2F/CHR pathway has provided a clearer explanation of how p53 downregulates a plethora of genes by activating p21^WAF1/CIP1^. It has also demonstrated the role of the p53-DREAM pathway in halting cell cycle progression in response to a number of stress signals including DNA damage as it leads to activation and stabilization of p53 (Horn and Vousden, 2007).

The key step in the p53-DREAM pathway is the upregulation of p21^WAF1/CIP1^ via direct binding of p53 to sites present in the p21^WAF1/CIP1^ promoter (Quaas et al., 2012). As p21^WAF1/CIP1^ is a CDKI it blocks phosphorylation of the pRB related pocket proteins, p107 and p130 as well as RB. In the unphosphorylated state p107 and p130 proteins bind to the MuvB core complex promoting the assembly of the repressive DREAM complex. Therefore, activation of p53 can shift the equilibrium from the activating MMB-FOXM1 complex to the repressive DREAM complex in a p21^WAF1/CIP1^ dependent manner (Quaas et al., 2012). During this stage, the DREAM complex shows parallel regulation along with pRB mediated regulation because lack of phosphorylation of pRB leads to the formation of repressive RB/E2F complexes (Dyson, 2016).

A recent study using meta-analyses of genome-wide studies has identified a catalog of more than 250 high confidence target genes of the p53-DREAM pathway (Engeland, 2017). This pathway controls genes important for cell functions spanning from the start (G1 phase) to the end of the cell cycle (M phase). Hence, p53 employs its master coordinator functions via the DREAM complex mediated mainly by p21^WAF1/CIP1^. Defects in the p53-pathway contribute to a loss of checkpoint control not only at the G1/S transition but at all checkpoints up to completion of the cell cycle. Activator E2F1-3 proteins bind to E2F elements for maximum expression of the genes involved in S phase whereas MMB-FOXM1 complex binds to CHR promoter elements to upregulate genes expressed in late G2 and M phase of cell cycle. The identification and detailed understanding of the DREAM complex has provided a clearer explanation for the precisely timed regulation of the G2/M cell cycle genes in addition to expression of late S phase genes by B-MYB-MuvB (MMB). This complements the well-established regulation of G1/S cell cycle genes by RB mediated repression of E2F TFs. In some senescent cells, senescence associated heterochromatin foci (SAHF) formation by pRB dependent reorganization of chromatin leads to silencing of E2F target genes (Narita et al., 2003). The stability of the cell cycle arrest during senescence is enforced by ROS production, secretion of cytokines and the heterochromatinization of E2F target genes. Derepression of retrotransposons and ribosome biogenesis defects have recently been discovered to be features of cell cycle arrest observed in senescent cells (Lessard et al., 2018; De Cecco et al., 2019).

There are striking chromatin alterations in senescent cells (Adams, 2007). Along with DDR (D’Adda Di Fagagna, 2008) and formation of PML bodies (Ferbeyre et al., 2000), SAHFs are the most prominent morphological change in chromatin (Narita et al., 2003). SAHF foci can be readily detected by DNA dyes such as DAPI and are characterized by enrichment of heterochromatin-associated repressive histone marks such as H3K9Me2, H3K9Me3 and chromatin reorganizing proteins such as heterochromatin protein (HP1), histone repressor A (HIRA) and anti-silencing function-1a (ASF1a), high mobility group A (HMGA) proteins, increased nuclear pore density and loss of linker histone H1 (Funayama et al., 2006; Chandra et al., 2012; De Cecco et al., 2013; Sadaie et al., 2013; Swanson et al., 2013; Salama et al., 2014; Chandra, 2016; Criscione et al., 2016; Boumendil et al., 2019; Chan and Narita, 2019). Since SAHFs are not seen in all senescent cells, it seems that they are cell type and stimulus dependent (Kennedy et al., 2010; Di Micco et al., 2011; Aird and Zhang, 2013; Criscione et al., 2016; Zirkel et al., 2018). Downregulation of Lamin B1, a key component of the nuclear lamina is a key feature of senescent cells, is known to trigger global and local chromatin changes impacting gene expression and promoting SAHF formation during senescence (Freund et al., 2012; Sadaie et al., 2013; Shah et al., 2013; Chandra et al., 2015).

Along with different mechanisms controlling cellular senescence, non-coding RNAs especially micro RNAs (miRNAs) have been demonstrated to play a key role in mediating cellular senescence alone or in conjunction with other effectors. Multiple studies have demonstrated that different miRNAs modulate the levels of key senescence effectors such as p53 (Hu et al., 2010; Burns et al., 2011; Xiao et al., 2011), p21^WAF1/CIP1^ (Borgdorff et al., 2010), p16^INK4A^ (Lal et al., 2008; Overhoff et al., 2014; Philipot et al., 2014), and SIRT1 (Suh, 2018; Baker et al., 2019; Barnes et al., 2019). miR 124, miR-34a/b/c, and miR-29a/b/c are upregulated in response to p53 activation and facilitate cellular senescence by downregulating survival and cell proliferation factors (Hermeking, 2010; Boon et al., 2013; Hu et al., 2014; Xu S. et al., 2019). A recent mRNA microarray and gene co-expression network analysis has revealed that most of the mRNAs that were downregulated by the activity of miRNA’s were involved in regulation of cell cycle progression (Xu S. et al., 2019). Fascinatingly, Ccna2 mRNA emerged as a common target of miR-29 and miR-124, which act as antagonists of p21^WAF1/CIP1^. This study showed that Ccna2 silencing significantly induced senescence whereas ectopic expression of exogeneous Ccna2 reversed the effect of miR-29 and miR-124, thereby substantially delaying cellular senescence and enhancing cell viability (Xu S. et al., 2019). This highlighted the important effect of Ccna2 in cellular senescence and identified a novel senescence regulator p53/miRNAs/Ccna2 pathway which acts independently of the canonical p53/p21^WAF1/CIP1^ pathway as the p53 responsive miRNAs were found to be significantly upregulated during senescence in p21^WAF1/CIP1^ deficient cells (Xu S. et al., 2019). Recently long non-coding RNAs (lncRNAs) which are more than 200 nucleotides long and capable of binding to DNA, RNA or proteins have been demonstrated to play a role in regulating senescence (Kim C. et al., 2017; Hu et al., 2018).

## The Senescence Associated Secretory Phenotype

Although senescent cells are in a growth arrested state, they remain metabolically active. Senescence does not only affect the events inside the cell but has the potential to affect the surroundings and communicate with neighboring cells by secreting a complex mixture of secreted factors which can alter the behavior of nearby non-senescent cells (Sun et al., 2018; Lopes-Paciencia et al., 2019; Mohamad Kamal et al., 2020). Cells undergoing senescence demonstrate significant changes in their secretome and exhibit a hyper secretory phenotype called the Senescence Associated Secretory Phenotype (SASP) (Coppé et al., 2010a) or Senescence-Messaging Secretome (SMS) (Kuilman and Peeper, 2009), one of the key hallmarks of senescence (Gorgoulis et al., 2019).

The main components of SASP include a plethora of soluble signaling factors, such as, pro-inflammatory cytokines, chemokines, growth modulators, angiogenic factors, proteases, bioactive lipids, extracellular matrix components, and matrix metalloproteinases (MMPs) (Coppé et al., 2010a; Freund et al., 2010; Acosta et al., 2013; Lopes-Paciencia et al., 2019). Even though multiple studies have identified the SASP components in different cell types, the exact composition of SASP remains elusive and is the topic of ongoing research. IGFBP3, IGFBP4, and IGFBP7 are key players of SASP that are suggested to be involved in mediating senescence by paracrine signaling (Wajapeyee et al., 2008; Severino et al., 2013; Özcan et al., 2016). The ability of IGFBP3 to induce senescence is regulated by tissue-type plasminogen activator inhibitor-1 (PAI-1) system (Elzi et al., 2012). PAI-1 is a critical downstream target of p53 involved in inducing replicative senescence via PI(3)K-PKB-GSK3β-cyclin D1 pathway (Kortlever et al., 2006). SASP plays a key role in mediating several of the pathophysiological effects of senescent cells and is therefore closely linked to its beneficial as well as deleterious effects (Rodier and Campisi, 2011). The SASP composition and strength varies substantially, depending on the inducer of senescence, duration of senescence, environment and cell type (Coppé et al., 2008, 2011; Maciel-Barón et al., 2016). The observed outcomes are both context dependent and cell type specific. However, NF-κB dependent pro-inflammatory factors are the key components of SASP with IL-6 and IL-8 being the most conserved and robustly expressed cytokines (Hardy et al., 2005; Davalos et al., 2010; Freund et al., 2010; Soto-Gamez and Demaria, 2017).

DNA damage, dysfunctional telomeres, genomic damage, epigenomic perturbation, mitogenic proliferative signals, oxidative stress or other senescence-inducing stimuli, all leading to prolonged DDR express SASP to different extents (Acosta et al., 2008; Kuilman et al., 2008; Rodier et al., 2009; Coppé et al., 2010b; Pazolli et al., 2012). In contrast, SASP is not detectable in cells where senescence is induced by ectopically expressing p21^WAF1/CIP1^ or p16^INK4A^, despite displaying other key senescence markers (Coppé et al., 2011). Therefore, DNA damage is an essential driver of SASP. However, a study by Freund et al. (2011), identified a novel canonical DNA damage response signaling independent mechanism that regulates SASP via p38MAPK (Freund et al., 2011). It was demonstrated that p38MAPK induced SASP mainly by inducing NF-κB activity (Freund et al., 2011). Similarly, induction of senescence by mitochondrial dysfunction presents a distinct secretory phenotype (Wiley et al., 2016).

Senescence associated secretory phenotype factors can reinforce and spread senescence by exerting their effects in both autocrine and paracrine fashion. Factors like IL-1A and IL-6 act in a cell-autonomous manner to reinforce the senescent state whereas many other SASP factors act by exerting non-cell-autonomous effects which enables alteration of the behavior of neighboring cells including manifestation of senescence in healthy, proliferation competent cells (Acosta et al., 2008, 2013; Nelson et al., 2012). This type of non-cell autonomous stable growth arrest is referred to as paracrine senescence. It was first demonstrated as a senescence bystander effect wherein senescent cells were capable of inducing DDR in neighboring cells (Nelson et al., 2012). This study suggested the involvement of reactive oxygen species (ROS) in paracrine senescence as it was observed that senescence occurred via gap junction-mediated cell to cell contact enabling transfer of ROS (Nelson et al., 2012, 2018). Autocrine and paracrine senescence along with immunosurveillance explain the accumulation of senescent cells observed upon aging and its detrimental effects.

Senescence associated secretory phenotype proteins can be secreted into the extracellular environment in a variety of ways. Many members are produced as soluble proteins which can be directly secreted, whereas others are initially expressed as transmembrane proteins that require ectodomain shedding for secretion (Stow and Murray, 2013). Enzymes like ADAM17 have been reported to be upregulated in OIS and cancer and are responsible for regulating the ectodomain shedding of many cell membrane-bound SASP factors (Effenberger et al., 2014; Morancho et al., 2015). Additionally, small exosome-like extracellular vesicles have recently emerged as key components of the senescent cell secretome to enable more distal functions, such as enhancing cancer cell proliferation (Takasugi et al., 2017), an intriguing topic requiring further investigation. SASP has been described as a temporally regulated dynamic program that can be divided into an initial rapid DDR-associated phase followed by an early self-amplification phase eventually leading to a late ‘mature’ phase (Malaquin et al., 2016).

### Mechanisms Involved in the Dynamic Regulation of the Senescence Associated Secretory Phenotype

**FIGURE 2:**
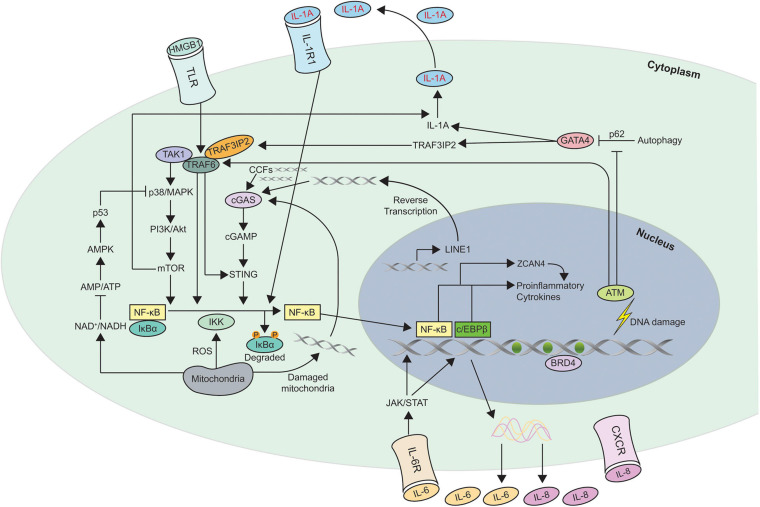
Schematic of the different mechanisms involved in Senescence Associated Secretory Phenotype (SASP) regulation. This figure shows the different pathways involved in regulating SASP. Most of the pathways converge to activate the transcription factors NF-κB and c/EBPβ in senescent cells. The autocrine feed forward signaling of different pro-inflammatory cytokines such as IL-1A, IL-6, and IL-8 is illustrated. ZCAN4 promotes the expression of inflammatory cytokines via NF-κB. TAK1 activates p38/MAPK, a kinase that subsequently engages PI3K/Akt/mTOR pathway. mTOR is capable of activating the NF-κB signaling directly as well as indirectly via IL-1A. GATA-4 links autophagy and DNA damage response to SASP via IL-1A and TRAF3IP2. NAD^+^, ROS, and DNA from damaged mitochondria are also involved in regulating SASP. Increase in transcription of LINE-1, a retrotransposable element in senescent cells facilitates accumulation of cDNA in the cytoplasm which leads to the activation of cGAS/STING pathway. In addition to LINE-1, CCFs, and DNA from damaged mitochondria are recognized by cGAS to generate cGAMP which subsequently activates STING to induce expression of SASP factors. The triggers for SASP activation can originate within the cell such as DNA damage, CCFs, cytosolic DNA or act on membrane receptors such as HMGB1, IL-1A, IL-6, and IL-8. Degradation of the inhibitor IκBα which sequesters NF-κB in cytosol, leads to nuclear translocation of NF-κB leading to expression of SASP genes. Recruitment of the chromatin reader BRD4 to newly activated super-enhancers adjacent to key SASP genes is needed for the SASP and downstream paracrine signaling. CCFs, cytoplasmic chromatin fragments; ROS, reactive oxygen species.

Multiple different nuclear and cytoplasmic factors such as DNA damage, cytoplasmic chromatin fragments (CCFs), transposable elements, and toll like receptors (TLR) have been shown to trigger SASP. Different pathways such as p38MAPK (Freund et al., 2011), JAK2/STAT3 (Hubackova et al., 2010; Xu et al., 2015b), inflammasome (Acosta et al., 2013), mTOR (Herranz et al., 2015; Laberge et al., 2015), phosphoinositide-3-kinase (PI3K) pathway (Bent et al., 2016; Zhang et al., 2018), HSP90 (Di Martino et al., 2018), non-coding RNAs (Bhaumik et al., 2009; Yap et al., 2010; Puvvula et al., 2014; Panda et al., 2017; Baker et al., 2019; Barnes et al., 2019), GATA4/p62-mediated autophagy (Kang et al., 2015), macroH2A1 and ATM (Chen et al., 2015) are all involved in the development and regulation of SASP. It is dynamically and temporally, regulated at multiple different levels such as chromatin modification, transcription, secretion, mRNA stability and translation. Figure 2 shows the different mechanisms involved in SASP regulation.

Most of the cascades involved in inducing and dynamically regulating SASP ultimately converge on the activation of two transcription factors, NF-κB and CEBPβ which are found to be enriched in the chromatin fractions of senescent cells (Acosta et al., 2008; Kuilman et al., 2008; Chien et al., 2011; Ohanna et al., 2011; Huggins et al., 2013). NF-κB and CEBPβ cooperatively control the transcription of key regulators of the inflammatory SASP proteins such as IL-1A, IL-6, and IL-8 which in turn positively regulate NF-κB and CEBPβ activity in an autocrine feed forward manner to enhance SASP signaling (Acosta et al., 2008; Kuilman et al., 2008; Orjalo et al., 2009; Rodier et al., 2009; Huggins et al., 2013; Zhang et al., 2018). IL-1A is a master regulator of SASP as ectopic expression of IL-1A can partially reproduce the inflammatory SASP characterized by expression of IL-1B, IL-6, IL-8, and CCL2 (Orjalo et al., 2009; Acosta et al., 2013). Cells which develop SASP can also transmit the phenotype to surrounding cells in a non-cell autonomous fashion via a complex secretory program orchestrated by the inflammasome; a multiprotein complex comprising caspase 1 and several adapter molecules (Schroder and Tschopp, 2010; Strowig et al., 2012), mainly by IL-1A and TGF-β mediating signaling both in cell culture and *in vivo* models of oncogene induced senescence (Acosta et al., 2013). Therefore, different components of SASP can reinforce the senescent state by amplifying or transmitting SASP via autocrine or paracrine signaling pathways (Kortlever et al., 2006; Acosta et al., 2008; Kuilman et al., 2008; Wajapeyee et al., 2008; Zhang et al., 2018). Moreover knock down of different SASP genes prevents senescence thereby highlighting the key role played by autocrine signaling mechanisms in regulating SASP and the senescent state (Acosta et al., 2008; Kuilman et al., 2008; Wajapeyee et al., 2008).

Senescence associated secretory phenotype gene expression can also be regulated by epigenetic changes. Persistent DNA damage leads to proteasome mediated degradation of G9a and GLP, two major histone H3K9 dimethyl transferases. This causes a global decrease in H3K9 dimethylation, an epigenetic mark for gene silencing and leads to induction of IL-6 and IL-8 (Takahashi et al., 2012). Other epigenetic regulators such as BRD4 (Tasdemir et al., 2016), MLL1 (Capell et al., 2016), HMGB2 (Guerrero and Gil, 2016), histone variant macroH2A1 (Chen et al., 2015), and GATA4 (Kang et al., 2015) are also involved in regulating SASP. In normal cells, GATA-4 bound to autophagy regulator, p62, is degraded by selective autophagy whereas induction of senescence in DNA damaged cells leads to suppression of autophagy and hence stabilization of GATA-4. The stabilized GATA-4 enhances SASP via TRAF3IP2 and IL-1A mediated NF-κB activation and establishes GATA4 as a separate branch of the senescence regulatory pathway independent of p53 and p16^INK4A^ for inducing SASP (Kang et al., 2015).

mTOR signaling has been demonstrated to be involved in regulating mammalian lifespan (Harrison et al., 2009). The exact mechanisms by which mTOR modulates aging are not clear but recent advances suggest a role in mediating SASP and cellular senescence. An mTOR dependent mechanism for regulating SASP at the post-transcriptional level has also been identified. Inhibition of mTOR by rapamycin suppresses the secretion of inflammatory cytokines by senescent cells (Herranz et al., 2015; Laberge et al., 2015). It also differentially regulates translation of IL-1A, the master regulator of SASP that subsequently engages IL-6/IL-8 (Orjalo et al., 2009; Laberge et al., 2015) and MAP kinase-activated protein kinase 2 (MAPKAP2) through 4EBP1, a translation repressor protein (Herranz et al., 2015). MAPKAP2 is known to phosphorylate and inhibit zinc finger protein 36L1 (ZFP36L1), an mRNA binding protein which binds to AU rich elements in the 5′-end of transcripts of proinflammatory SASP components and target them for degradation (Herranz et al., 2015) thereby enabling mTOR to indirectly regulate SASP by regulating mRNA stability. Hence, mTOR interacts with the p38MAPK signaling pathway as MAPKAP2 is a downstream target of p38MAPK. In cells undergoing OIS, spatial integration of mTOR and autophagy which couples protein synthesis and degradation boosts the production and secretion of SASP components in a distinct cellular compartment at the trans site of the Golgi apparatus, the TOR-autophagy spatial coupling compartment (TASCC) (Narita et al., 2011; Young et al., 2013; Herranz et al., 2018).

Last year yet another mechanism of SASP regulation demonstrating a novel role of NAD+ metabolism involving HMGA-NAMPT-NAD^+^ signaling axis in regulating SASP was identified (Nacarelli et al., 2019). HMGA proteins modify chromatin structures to regulate senescence (Narita et al., 2006). Nicotinamide phosphoribosyl transferase (NAMPT) catalyzes the rate limiting step in the NAD salvage pathway from nicotinamide (NAM) (Garten et al., 2015; Verdin, 2015). It was found that in OIS, HMGA1 plays an instrumental role in upregulating NAMPT through an enhancer element which in turn promotes inflammatory SASP in response to an increased NAD^+^/NADH ratio (Nacarelli et al., 2019). In accordance with this, inhibition of HMGA1 and NAMPT suppresses OIS initiation showing that HMGA-NAMPT-NAD^+^ signaling promotes proinflammatory SASP through the NAD^+^ mediated suppression of AMPK. AMPK suppresses p53 mediated inhibition of p38MAPK to enhance NF-κB activity to promote expression of proinflammatory SASP proteins. Thus HMGA-NAMPT-NAD^+^ mediated expression of proinflammatory SASP is independent of CEBPβ activity. Enhanced glycolysis and mitochondrial respiration have been shown to promote NAD^+^ dependent proinflammatory SASP. Taken together, the ratio of NAD^+^/NADH regulated by NAMPT which acts downstream of HMGA1 governs the strength of proinflammatory SASP. This suggests that an increase in the ratio of NAD^+^/NADH is capable of converting a low proinflammatory SASP into a high proinflammatory SASP.

Recently TLR, an innate immune receptor which recognizes pathogen-associated molecular patterns (PAMPs) and damage associated molecular patterns (DAMPs), was shown to trigger induction of SASP (Davalos et al., 2013; Kawasaki and Kawai, 2014; Loo et al., 2017, 2020; Hari et al., 2019). New key SASP components, acute-phase serum amyloids A1 and A2 (A-SAAs), which act as senescence associated DAMPs and induce SASP through TLR2 after oncogenic stress have been identified (Hari et al., 2019). Lipoteichoic acid (LTA), a component of the cell wall of gram positive gut microbiota is recognized by TLR2 and induces expression of SASP components creating a tumor promoting micro environment that promotes development of obesity associated hepatocellular carcinoma (Loo et al., 2017). In accordance with this, HMGB1 secreted by senescent fibroblasts is recognized by TLR4, followed by increase in SASP secretion (Davalos et al., 2013). These findings establish the critical role played by innate immune sensing mechanisms in regulating senescence.

Recent studies have revealed that cytoplasmic chromatin fragments and transposable elements can stimulate cyclic GMP–AMP synthase linked to stimulator of interferon genes (cGAS-STING) pathway and regulate SASP both *in vitro* and *in vivo* where senescence was induced by different stimuli (Dou et al., 2017; Glück et al., 2017; Yang et al., 2017; Li and Chen, 2018). The mechanisms implicated in the accumulation of cytoplasmic DNA in senescent cells include compromised nuclear integrity due to loss of the nuclear lamina protein, Lamin B1 (Dou et al., 2017) and downregulation of cytoplasmic DNAases such as DNAase 2 and TREX1 (Takahashi et al., 2018) frequently observed in senescent cells. De Cecco et al. (2019) reported derepression of long-interspersed element-1 (L1 or LINE-1), the only human retrotransposable element capable of autonomous retrotransposition, in senescent cells. This activation of L1 which is mediated by TREX1, RB1 and FOXA1 leads to accumulation of cDNA in the cytoplasm as L1 possesses high reverse transcriptase activity. The accumulated cDNA has been demonstrated to trigger cGAS/STING signaling pathway, leading to production of SASP factors (De Cecco et al., 2019).

cGAS a 522 amino acid protein, is a cytosolic DNA sensor that activates innate immunity upon sensing aberrant double stranded (ds) DNA molecules irrespective of the source. In the presence of ATP and GTP, cGAS catalyzes the production of 2′3′ cyclic GMP-AMP (cGAMP) which stimulates the adaptor protein, STING. STING recruits TANK-binding kinase 1 (TBK1) and IK-B kinase (IKK) (Ablasser et al., 2013; Diner et al., 2013; Gao et al., 2013; Sun et al., 2013, 2018; Wu et al., 2013; Zhang et al., 2013; Ablasser and Gulen, 2016; Chen et al., 2016). TBK1 phosphorylates the transcription factor IRF3, leading to its translocation from cytosol to the nucleus where it activates the transcription of type-I interferons such as IFN-β (Sun et al., 2013, 2018; Loo et al., 2020). IKK activates transcription factor NF-κB to induce expression of pro-inflammatory cytokines such as IL-6 and IL-8 (Barber, 2015; Sun et al., 2018; Loo et al., 2020). Hence, the two key downstream pathways activated downstream of cGAS-STING involve activation of type-I interferon and NF-κB.

Recently, a novel role of cGAMP as a soluble extracellular immunotransmitter produced and secreted by malignant cells has been identified. Using a genome wide CRISPR interference screen, SLC19A1, was identified as the first known major importer of cGAMP, which is taken up by host cells to activate intracellular STING pathway to elicit an antitumor immune response. This suggests a highly likely possibility that senescent cells secrete cGAMP via SLC19A1 to promote a paracrine innate immune response (Luteijn et al., 2019; Ritchie et al., 2019). Interestingly, cGAS/STING pathway was recently shown to regulate the induction of TLR2 and A-SAAs in oncogene induced senescence, suggesting that TLR2 signaling occurs downstream of cGAS/STING and is mainly regulated by NF-κB (Hari et al., 2019).

Loss of cGAS compromised senescence due to reduced SASP in different *in vivo* models and also accelerated the spontaneous immortalisation of mouse embryonic fibroblasts thereby highlighting the crucial role of the cGAS/STING pathway in tumor suppression due to immune-mediated clearance of premalignant cells (Dou et al., 2017; Glück et al., 2017; Umbreit and Pellman, 2017; Yang et al., 2017). Identification of the cGAS-STING pathway has shown that nuclear genomic DNA not only acts as a stable nuclear entity that encodes genetic information but can also serve to act as a ‘danger-signal’ when in the cytoplasm and alarm the immune system by inducing the proinflammatory SASP pathway.

Most of the regulatory mechanisms reviewed here have detailed the control of the expression of pro-inflammatory arm of SASP which is shown to be highly conserved among different forms of cellular senescence such as replicative, irradiation-induced, and OIS. However, the pro-inflammatory arm is not the only subset of SASP as its composition is highly variable and heterogeneous (Wiley et al., 2017). Table 1 details the SASP factors involved in different senescence contexts. This suggests the possibility that different SASP factors are regulated by different mechanisms. Recent findings have indicated dynamic signaling by NOTCH1, a transmembrane receptor, as a crucial regulator of SASP composition which governs the transition between the inflammatory secretome and TGF-β enriched secretome. In OIS, NOTCH1 activity correlates with the expression of TGF-β1 and TGF-β3 factors and inversely corelates with expression of typical inflammatory cytokines such as IL1A, IL-6, and IL-8. This NOTCH1 mediated suppression of inflammatory cytokines is manifested primarily by the repression of c/EBPβ mediated transcription and not NF-κB. In accordance with this, NOTCH1 inhibition has been shown to facilitate the upregulation of proinflammatory SASP components (Hoare et al., 2016; Ito et al., 2017). This highlights the novel role of NOTCH1 as a temporospatial controller of SASP composition dictating the functional balance between two distinct secretomes; the pro-inflammatory and the TGF-β enriched immunosuppressive secretome. Another cause of senescence growth arrest, mitochondrial dysfunction, different from senescence due to genotoxic stress has recently been identified (Wiley et al., 2016). Mitochondria normally oxidize NADH to NAD^+^ and mitochondrial dysfunction decreases the NAD^+^/NADH ratio mostly in the cytosol, leading to activation of 5′ adenosine monophosphate activated protein kinase (AMPK), resulting in p53 activation and mitochondrial dysfunction associated senescence (miDAS) (Wiley et al., 2016). The key feature of miDAS is the distinct secretory phenotype, different from canonical SASP caused by genotoxic stress. This SASP lacks the canonical IL-1 mediated inflammatory components but comprises interleukin (IL)-10, tumor necrosis factor alpha (TNF-α) and chemokine (C-C motif) ligand 27 (CCL27) (Wiley et al., 2016).

**TABLE 1:** Selected list of SASP factors involved in different senescence contexts.

**Senescence**	**SASP factors**
Replicative senescence	Angiogenin, bFGF, COX-2, CXCR2, Eotaxin-3, Fas, FGF-7, Fibronectin, GM-CSF, GROα,β,γ, HCC-4, HGF, ICAM-1, IFN-1, IGFBP1, IGFBP2, IGFBP3, IGFBP4, IGFBP5, IGFBP6, IL-1A, IL-1B, IL-6, IL-7, IL-8, IL-11, IL-15, IL-13, Leptin, MCP-1, MCP-2, MCP-4, MIF, MIP-1α, MIP-3α, MMP-1, MMP-2, MMP-3, MMP-10, Osteoprotegerin, PAI-1, PAI-2, PGE-2, PIGF, SCF, sgp130, sTNF RI, sTNF RII, TGFβ, TIMP-2, tPA, TRAIL-R3, uPA, uPAR, WNT2.
DNA-damage-induced senescence	Acrp30, Amphiregulin, Angiogenin, bFGF, BTC, CTACK, EGF-R, ENA-78, Eotaxin-3, Fas, FGF-7, GCP-2, GDNF, GITR, GM-CSF, GROα,β,γ, HCC-4, HGF, I-309, ICAM-1, IGFBP1, IGFBP2, IGFBP3, IGFBP4, IGFBP5, IGFBP6, IL-1A, IL-1B, IL-6, IL-6R, IL-7, IL-8, IL-11, IL-13, IL-15, IL-1R1, IL-2R-α, I-TAC, Leptin, MCP-1, MCP-2, MCP-4, MIF, MIP-1α, MIP-3α, MMP-1, MMP-2, MMP-3, MMP-10, MMP-12, MMP-13, MMP-14, MSP-a, Oncostatin M, Osteoprotegerin, PDGF-BB, PIGF, RANTES, SCF, SDF-1, sgp130, sTNF RI, sTNF RII, Thrombopoietin, TIMP-1, TIMP-2, tPA, TRAIL-R3, uPA, uPAR, VEGF
Oncogene-induced senescence	Angiogenin, AREG, A-SAA, bFGF, BLC, CCL1, CCL2, CCL7, CCL20, COX2, CXCR2, CXCL5, CXCL6, ENA-78, Eotaxin-3, GCP-2, G-CSF, GITR, GMCSF, GROα,β,γ, HCC-4, HGF, I-309, ICAM-1, IFN-1, IFN-γ, IGFBP-4, IGFBP-6, IGFBP7, IL-1A, IL-1B, IL-6, IL-6R, IL-7, IL-8, IL-13, I-TAC, LIF, MCP-1, MCP-2, MCP-4, MIF, MIP-1α, MIP-3α, MMP1, MMP3, MMP10, NAP-2, Oncostatin M, Osteoprotegerin, PAI-1, PGE-2, PIGF, SDF-1, sgp130, sTNF RI, t-PA, TIMP-1, TIMP-2, uPAR, VEGF
Therapy-induced senescence	AREG, CXCL8, IL1A, IL-1B, IL-6, MMP2, MMP3, PAI-1, SPINK1, t-PA, WNT16B
Mitochondrial dysfunctional associated senescence	Lacks IL-1-dependent factors but includes IL-10, CCL-27, TNF-α,
SASP factors involved in development	CD-44, CSF-1, FGF, IGFBP-5, WNT5A
SASP factors involved in wound healing	CCL-2, CCL-5, CCN1, CCN2, PAI-1, PDGF-AA, VEGF

*Data are based on Kuilman and Peeper (2009), Coppé et al. (2010a), Freund et al. (2010), Wiley et al. (2016), and Lopes-Paciencia et al. (2019).*

*AREG, amphiregulin; A-SAA, acute-phase serum amyloids A1 and A2; COX2, cyclooxygenase 2; CXCR, CXC chemokine receptor; bFGF, basic fibroblast growth factor; EGF, endothelial growth factor; GMCSF, granulocyte-macrophage colony stimulating factor; GRO, growth-related oncogene; HGF, hepatocyte growth factor; ICAM, intercellular adhesion molecule; IFN, interferon; IGFBP, insulin-like growth factor binding protein; IL, interleukin; MCP, membrane cofactor protein; MMP, matrix metalloproteinase; PAI, plasminogen activator inhibitor; PDGF, platelet-derived growth factor; PGE2, prostaglandin E2; PIGF, placental growth factor; SCF, stem cell factor; SDF, stromal cell derived factor; sTNFR, soluble tumor necrosis factor receptor; TGFβ, transforming growth factor β; TIMP, tissue inhibitor of metalloproteinases; TNF, tumor necrosis factor; t-PA, tissue-type plasminogen activator; TRAIL, tumor necrosis factor related apoptosis-inducing ligand; uPA, urokinase-type plasminogen activator; uPAR, uPA receptor; VEGF, vascular endothelial growth factor.*

Acute stress-associated phenotype (ASAP), characterized by expression of IL-6 and Timp-1, represents an early phase of cellular response observed immediately after exposure to cytotoxic agents. In contrast, in most cells, SASP develops gradually over a course of 5–10 days after senescence markers are detected (Gilbert and Hemann, 2010, 2011; Sun et al., 2018). ASAP occurs in the context of PI3K/Akt/mTOR signaling suppression independently of DDR and mTOR signaling, further distinguishing it from canonical SASP (Bent et al., 2016). DNA damaged human stromal cells, transition from transient ASAP to chronic SASP during acute DDR; this is mediated by expression of Zscan4 enhanced by the ATM-TRAF6-TAK1 axis (Zhang et al., 2018). Interestingly following DNA damage, TAK1, a crucial kinase involved in ASAP, eventually activates PI3K/Akt/mTOR and p38MAPK pathways to sustain persistent SASP signaling (Zhang et al., 2018). Therefore, the heterogeneous nature of SASP is due to the involvement of multiple different signaling molecules required for manifesting SASP in a stressed setting. Senescent cells can communicate with the surroundings through juxtacrine NOTCH/JAG1 signaling (Hoare et al., 2016; Ito et al., 2017) or ROS secretion (Kuilman et al., 2010; Nelson et al., 2012, 2018) or cargo transfer by formation of cytoplasmic bridges (Biran et al., 2015) or release of extracellular vesicles such exosomes (Lehmann et al., 2008; Takasugi et al., 2017).

### Functions of the SASP

Collectively, the SASP secretome is the characteristic of senescent cells that confers most of its biological effects both the beneficial as well as deleterious effects, and therefore is the key regulator of normal physiology and pathology associated with cellular senescence. The composition of SASP is heterogenous; the functions are also quite diverse and depend on the genetic context of the cells exposed to SASP and the neighboring environment (Table 1). The effect of autocrine and paracrine signaling of different SASP factors in a specific context are pleiotropic which explains the paradoxical roles for cellular senescence. For example, IL-6 and IL-8 are two main SASP components which have been shown to play both positive and negative roles in different biological processes such as wound healing, tissue repair and tumor progression (Coppé et al., 2010a; Demaria et al., 2014).

Findings so far suggest that the SASP might have originated to help damaged senescent cells to communicate with neighboring cells about their compromised state and initiate tissue repair and regeneration by stimulating the nearby progenitor cells, or to stimulate the immune system to promote their immune clearance (Xue et al., 2007; Kang et al., 2011; Iannello et al., 2013; Tasdemir et al., 2016; von Kobbe, 2018; Mohamad Kamal et al., 2020). Senescence may also have evolved as an exaptation of developmental senescence or as a viral defense mechanism. SASP components control a multitude of functions and play key beneficial physiological roles such as: accelerated wound healing by secreting factors such as PDGF-AA and CCN1 (Jun and Lau, 2010; Demaria et al., 2014), promoting stemness and tissue plasticity in response to damage to maintain tissue homeostasis (Ritschka et al., 2017; Taguchi and Yamada, 2017), embryonic development (Muñoz-Espín et al., 2013; Storer et al., 2013), fibrotic scar degradation (Krizhanovsky et al., 2008; Lujambio et al., 2013), and tumor suppression (Lujambio et al., 2013; Rao and Jackson, 2016). SASP mediated autocrine and paracrine signaling that reinforces senescence contribute to tumor suppressive functions of SASP by limiting the proliferation of cells at risk followed by immune clearance of the premalignant cells (Acosta et al., 2008, 2013; Kuilman et al., 2008; Wajapeyee et al., 2008; Kang et al., 2011; Hubackova et al., 2012; Nelson et al., 2012; Toso et al., 2014). However, the factors that determine the balance between repair, regeneration and senescence, in response to damage, require further examination.

Recent discoveries suggest that the deleterious effects of SASP overshadow its beneficial properties. Inflammatory SASP components and accumulation of immature immunosuppressive myeloid cells in solid tumors promote tumorigenesis by driving cell migration, growth, invasion, angiogenesis and eventually metastasis (Krtolica et al., 2001; Coppé et al., 2006; Yoshimoto et al., 2013; Di Mitri et al., 2014; Eggert et al., 2016; Demaria et al., 2017; Kim Y.H. et al., 2017; Chen et al., 2018). This demonstrates the multifaceted interaction between SASP, immune cells and cancer, in accordance with Eggert et al. (2016), where in the initial stages, SASP mediated recruitment of immature myeloid cells (iMC) which when differentiated into macrophages clear the pre-malignant senescence cells to prevent cancer initiation. On the other hand, in later stages the tumor cells block the maturation of the accumulated iMC which eventually promote the growth of established hepatocellular carcinoma by inhibiting NK cell functions (Eggert et al., 2016). SASP also mediates the harmful effects of senescent cells which accumulate upon chemotherapy treatment as their chronic presence promotes local and systemic inflammation. Elimination of therapy-induced senescent cells can prevent cancer recurrence (Demaria et al., 2017). Recently, another SASP factor amphiregulin (AREG) has been shown to drive cancer resistance via EFGR pathway and augment malignancy (Xu Q. et al., 2019). Multiple studies have shown that most of the age-related pathologies stem from low level chronic inflammation referred to as inflammaging or sterile inflammation which can also result in premature aging (Franceschi et al., 2007; Chung et al., 2009; Franceschi and Campisi, 2014; Jurk et al., 2014). Therefore, SASP mediated autocrine and paracrine signaling may explain how a relatively small number of senescent cells can bring about durable, local and systemic effects *in vivo*, which promote chronic diseases and age-associated functional decline (Allavena et al., 2008; Coppé et al., 2010a; Lecot et al., 2016; Contrepois et al., 2017). Taken together, SASP components have the potential to alter different cellular processes within the microenvironment wherein a chronic SASP causes negative outcomes whereas a short-lived transient SASP is beneficial.

Senescence associated secretory phenotype is nonspecific, context dependent and highly heterogeneous which can be regulated at multiple different levels. However, the difficulty in the identification of a general regulatory mechanism restricts its utility as an unequivocal marker for senescence as no unique form of SASP is known to exist (Coppé et al., 2010a; Hernandez-Segura et al., 2017; Sun et al., 2018). Nevertheless studying the composition of SASP can be very helpful in defining different senescence programs and their context and potentially be used to target SASP for therapeutic purposes (Lecot et al., 2016). For example, the presence of different MMPs and growth factors like VEGF and PDGF-A indicate the involvement of senescent cells in and tissue repair and wound healing (Jun and Lau, 2011; Demaria et al., 2014) whereas, age-related or therapy-induced senescent cells are mainly linked with secretion of inflammatory factors (Baker et al., 2016; Demaria et al., 2017). Analysis of individual cells following induction of cellular senescence by single cell RNA sequencing has revealed surprisingly significant cell to cell variation in SASP gene expression (Wiley et al., 2017). Therefore, it is possible that single cell profiling may allow us to understand which particular SASP component drives a particular function in a specific context *in vivo*.

## The DNA Damage Response (DDR)

Different intrinsic (telomere attrition, hyperproliferation, oxidative damage) and extrinsic (γ-irradiation, ultraviolet radiations, chemotherapeutic drugs) stimuli lead to persistent DDR signaling which results in irreparable DNA damage and induce cellular senescence (D’Adda Di Fagagna, 2008; Fumagalli et al., 2012). It has been observed that a single unresolved DNA DSB is capable of inducing senescence (Di Leonardo et al., 1994). DDR machinery in human fibroblasts senses an uncapped, double stranded chromosome free end exposed due to progressive telomere shortening in replicative senescence to initiate a DDR (D’Adda Di Fagagna et al., 2003). Human somatic cells lack the catalytic subunit of telomerase at a level sufficient to maintain telomeres after repeated cell division which results in shortening of telomeres due to the end replication problem (Shay and Wright, 2019). During oncogene induced senescence, oncogene activation initially triggers a hyperproliferative phase which induces cellular senescence. The mitotic signals increase the usage of origins of replication resulting in stalled replication forks and accumulation of genomic damage that eventually activates the DDR (Bartkova et al., 2006; Di Micco et al., 2006; Halazonetis et al., 2008; Gorgoulis and Halazonetis, 2010). Both DDR and ARF tumor suppressor mechanisms are involved in mediating oncogene induced senescence (OIS); DDR is more sensitive and requires less oncogenic load than ARF (Evangelou et al., 2013; Gorgoulis et al., 2018). DDR in replicative senescence is dependent on the telomere length whereas in OIS it is not due to telomere length, even though telomere dysfunction is associated with OIS (D’Adda Di Fagagna et al., 2003; Suram et al., 2012). Both telomeric and non-telomeric DNA damage have been shown to play equivalent roles in triggering senescence (Nakamura et al., 2008). Prolonged DDR signaling caused directly or indirectly by DNA DSBs can enforce senescence growth arrest mostly regulated via the p53/p21^WAF1/CIP1^ pathway (Fumagalli et al., 2012).

DNA damage such as single-strand and double-strand breaks activates DDR, which is a classical, evolutionarily conserved, robust response to damaged DNA. Normally cells are able to deal with DNA damage, but cells must undergo either apoptosis or senescence if the damage is irreparable, to prevent progression of damaged cells. The choice between apoptosis and senescence depends on the extent and the duration of the DNA damage signaling. It has now been suggested that prominent short-term DNA damage activates apoptosis whereas prolonged mild DNA damage induces cellular senescence (Petrova et al., 2016).

Irrespective of the stimuli driving the DDR, classical DDR mainly involves the p53/p21^WAF1/CIP1^ tumor suppressor pathway. Multiple different DNA damage sensors such as replication protein A (RPA) (Zou and Elledge, 2003) and the RAD9-RAD1-HUS1 (9-1-1) (Weiss et al., 2002) complex detect exposed single-stranded breaks and MRE11-RAD50-NBS1 (MRN) (Stracker et al., 2004; Moreno-Herrero et al., 2005) complex detect DNA double-stranded breaks and recruit the upstream protein kinases ataxia telangiectasia mutated (ATM) and ataxia telangiectasia and RAD3-related (ATR) to the site of damage (D’Adda Di Fagagna, 2008). Although both ATM and ATR are activated upon DNA damage, they have distinct DNA specificities; ATM gets activated predominantly by DSBs whereas ATR in addition to double strand breaks responds to a broad spectrum of DNA damage such as genotoxic stress caused by DNA replication stress initiated by oncogenes (Maréchal and Zou, 2013). Once at the site of damage ATM and ATR amplify the DDR signal by phosphorylating other DNA damage mediator proteins, such as histone H2AX to form γ-H2AX which aids in the assembly of other specific DNA repair complexes, forming nuclear foci that are stable sites of dynamic accumulation of different DDR proteins (Lukas et al., 2003). Dynamic changes in histone modification such as histone methylation are also critical for regulating DNA double-strand break (DSB) repair by activating ATM kinase which also contributes to the formation of transient repressive chromatin structures which serve to stabilize the damaged chromatin and promote assembly of DSB-signaling proteins (Ayrapetov et al., 2014).

CHK1 and CHK2 are the downstream diffusible kinases which act far from the site of DNA damage and propagate the damage signal by phosphorylating the final effector substrates such as p53. Phosphorylation of p53 on Serine-20 by CHK2 leads to a reduction in binding affinity of the E3 ubiquitin ligase MDM2 to p53, leading to an increase in p53 levels. p53 is also phosphorylated at Serine-15 directly by ATM (Chehab et al., 1999). CHK1 negatively regulates CDC25, a dual-specificity protein phosphatase which promotes the G2 to M transition, by phosphorylating Serine-216 leading to a G2 growth arrest (Peng, 1997). Phosphorylated p53 upregulates the expression of p21^WAF1/CIP1^, a potent universal CDKI leading to cell cycle arrest (Sulli et al., 2012).

In addition to the role of DDR in manifesting senescence associated cell cycle arrest, DDR signaling also mediates SASP by inducing NF-κB activation. The genotoxic stress sensor ATM and PARP-1 stimulate NF-κB transcriptional activity (Stilmann et al., 2009). Chemotherapeutic drugs or oxidative stress induced DNA damage engage PARP-1/ATM/NF-κB signaling cascade to induce senescence in melanoma and non-melanoma cells (Ohanna et al., 2011). These senescent cells develop a PARP-1 and NF-κB associated secretome (PNAS) containing chemokine CCL2 along with other SASP factors thereby augmenting the invasiveness of melanoma cells which might have escaped senescence. Blocking PARP-1, ATM, or NF-κB in melanoma cells prevents the secretion of chemokine CCL2 thereby restricting the deleterious pro-invasive properties of the inflammatory SASP mediated by PNAS (Ohanna et al., 2011).

As most of the senescence-inducing stimuli eventually impinge directly or indirectly on DNA, persistent DDR signaling is a characteristic feature of many senescent cells. DDR associated features such as DNA damage foci which can be detected by immunostaining of γ-H2AX; DNA segments with chromatin alterations reinforcing senescence (DNA-SCARS) (Rodier et al., 2011) and telomere-dysfunction induced foci (TIF) (Herbig et al., 2006) or phosphorylated p53, can be used as markers for cellular senescence. However, despite this, DDR markers have a limited potential for identifying senescent cells *in vivo* since DDR independent mechanisms are also capable of inducing senescence via p53/p21^WAF1/CIP1^ pathway (Alimonti et al., 2010; Freund et al., 2011; Muñoz-Espín et al., 2013; Storer et al., 2013; Salama et al., 2014). DDR can also be activated by other DNA-damaging stimuli which do not lead to the development of the senescent state but are rather involved in physiological non-pathological settings or are in the process of responding to a transient repairable DNA damage.

## Discussion

Here we have reviewed the different mechanistic pathways as well as the various mediators which underlie the finite proliferative of normal somatic cells and how entry into senescence leading to a stable cell cycle arrest and secretion of the SASP proteins is regulated. Although bypassing senescence and acquiring a limitless replicative potential is a key event required for malignant transformation, the underlying signaling pathways and the basis for the stability of the growth arrest are poorly understood (Hanahan and Weinberg, 2011). A greater understanding is therefore essential if we are to prevent tissue dysfunction without increasing the risk of developing cancer. There is also abundant room for further progress in better understanding the mechanisms underlying the short-lived, transient senescence which benefits tissue development, regeneration and repair as this is less well-characterized in comparison to the deleterious effects of stable senescence.

One of the key stumbling blocks in the field of senescence is the lack of a single, universal, robust, biomarker that allows identification of senescent cells with high sensitivity and specificity and is capable of differentiating them from terminally differentiated, quiescent, and other non-dividing cells. Growth arrest is a key feature which can be readily demonstrated *in vitro* using colony-formation assays or by BrdU/EdU-incorporation assays that measure DNA synthesis (Cavanagh et al., 2011; Crane and Bhattacharya, 2013; Mead and Lefebvre, 2014; Adan et al., 2016; Bhaskara, 2016). However, DNA synthesis measurement is not totally specific since DNA repair may still be active. Measuring the expression levels of CDKIs p16^INK4A^ and p21^WAF1/CIP1^ are key to detecting cell cycle arrest but are not expressed persistently particularly p21^WAF1/CIP1^ by senescent cells (Herbig et al., 2004; Da Silva-Álvarez et al., 2019). Accumulation of high levels of p16^INK4A^ is required to maintain the senescent state by preventing RB inactivation enabling it to be extensively used as a marker for senescence in most normal untransformed cells and tissues (Hara et al., 1996; Sharpless and Sherr, 2015; Wiley et al., 2017). However, p16^INK4A^ is also expressed in non-senescent cells and cells that are transiently arrested, and senescence can also occur independently of p16^INK4A^ coupled with the lack of specific antibodies limits its use as a biomarker for senescence (Sharpless and Sherr, 2015; Herranz et al., 2018).

Due to the heterogeneous and dynamic nature of senescence, there is currently no single totally reliable biomarker (Carnero, 2013). Recently a multi-marker, three-step workflow which allows accurate detection of senescent cells has been proposed (Gorgoulis et al., 2019). The first step includes assessing senescence-associated-beta-galactosidase (SA-β-gal) activity and/or lipofuscin accumulation (GL-13 or SBB). The second step examines frequently observed markers of senescent cells including transcriptional signatures linked to the cell-cycle arrest and SASP such as increased expression of the cyclin-dependent kinase inhibitors and a subset of SASP genes, along with decreased expression of proliferation markers such as cyclins, CCNA2 and CCNE2 and LMNB1. The third step consists of identification of factors that are anticipated to be altered in the specific context. Single-cell transcriptome and proteome profiling of tissues along with development of sophisticated high-throughput methods and machine learning tools will be key to understanding the nature of senescent cells and may aid in identifying potential therapeutic approaches (Vougas et al., 2019). To help with the identification of genes associated with senescence a novel database SeneQuest^1^ has been established (Gorgoulis et al., 2019).

A recent study by Martínez-Zamudio et al. (2020), revealed links between enhancer chromatin, transcription factor recruitment, and senescence competence. They demonstrated that a hierarchical transcription factor network defines the senescence transcriptional program and identified activator protein 1 (AP-1) as a master regulator that drives the transcriptional program of senescent cells thereby revealing promising pathways with therapeutic implications for modulation of senescence *in vivo*.

Accumulating evidence has demonstrated that both anti-senescence and pro-senescence therapies could be beneficial depending on the context (Myrianthopoulos et al., 2019; van Deursen, 2019). Pro-senescence therapies help limit damage by restraining proliferation and fibrosis during carcinogenesis and active tissue repair whereas anti-senescence agents enable elimination of accumulated senescent cells to restore tissue function, and potentially aid organ rejuvenation (McHugh and Gil, 2018; Gorgoulis et al., 2019). It has been found that cells which escape from senescence post-chemotherapy re-enter the cell cycle, are highly aggressive, chemo-resistant, and exhibit stem cell characteristics and can contribute to cancer recurrence (Milanovic et al., 2018; Saleh et al., 2019). Since several therapeutic modalities trigger senescence in tumors, it is important to decipher the mechanisms involved in the escape from senescence as a more detailed understanding may allow the development of better therapies and also help to reduce the off-target effects contributing to unwanted toxicity.

A thorough understanding of SASP regulation is required to exploit it for therapeutic purposes. There is a growing need for further research to investigate how the different signaling pathways regulating SASP such as p38MAPK, mTOR, GATA4, TAK1, cGAS/cGAMP/STING are interconnected and how SASP manifests the age-related pathologies. Inhibition of SASP without perturbing the stable growth arrest would allow reduction of the deleterious effects while maintaining tissue homeostasis and other physiological roles. However, targeting SASP for therapeutic purposes has to be undertaken with great care since it has both beneficial and deleterious roles due to the plethora of components.

Identification of key SASP factors secreted by senescent cells in aged tissues and residual tumors in the post-treatment period might have potential as biomarkers for real-time medical surveillance. The advent of powerful genetic and pharmacological tools to dissect the relationship between accumulated senescent cells and aging should improve our understanding of how accumulated senescent cells lead to age associated decline. The detailed kinetics of accumulation of senescent cells during the lifetime of an organism, remain to be established. It is important to note that despite the link between senescent cells and organismal aging, senescence and aging should not be considered synonymous as cells can undergo senescence due to a multitude of stimuli in addition to telomere shortening irrespective of organismal age. Identification of factors which control or determine the balance between senescence, regeneration and repair require investigation.

A greater in-depth understanding of the underlying mechanisms which regulate senescence will provide promising translational opportunities to develop new therapeutic approaches which minimize the detrimental consequences of senescence. Targeting senescence using senolytics to selectively eliminate senescent cells or modulate SASP using small molecules or antibodies will not only aid in treatment of senesce related diseases but may contribute toward improving the health span of individuals.

## Author Contributions

RK and PJ have contributed equally in the preparation of manuscript. Both authors contributed to the article and approved the submitted version.

## Conflict of Interest

The authors declare that the research was conducted in the absence of any commercial or financial relationships that could be construed as a potential conflict of interest.

## Footnotes

1 http://Senequest.net
